# Time-varying intensity of ventilatory inefficiency and mortality in patients with acute respiratory distress syndrome

**DOI:** 10.1186/s13613-025-01427-1

**Published:** 2025-01-13

**Authors:** Lianlian Jiang, Hui Chen, Wei Chang, Qin Sun, Xueyan Yuan, Zongsheng Wu, Jianfeng Xie, Ling Liu, Yi Yang

**Affiliations:** Jiangsu Provincial Key Laboratory of Critical Care Medicine, Department of Critical Care Medicine, Zhongda Hospital, School of Medicine, Southeast University, No. 87, Dingjiaqiao Road, Gulou District, Nanjing, 210009 People’s Republic of China

**Keywords:** Acute respiratory distress syndrome, Mechanical ventilation, Arterial carbon dioxide pressure, Ventilatory ratio

## Abstract

**Background:**

The association between bedside ventilatory parameters—specifically arterial carbon dioxide pressure (PaCO_2_) and ventilatory ratio (VR)—and mortality in patients with acute respiratory distress syndrome (ARDS) remains a topic of debate. Additionally, the persistence of this association over time is unclear. This study aims to investigate the relationship between 28-day mortality in ARDS patients and their longitudinal exposure to ventilatory inefficiency, as reflected by serial measurements of PaCO_2_ and VR.

**Methods:**

We conducted a secondary analysis of four randomized controlled trials (FACTT, ALTA, EDEN, and SAILS) from the ARDS Network. All included patients were intubated and received mechanical ventilation. Patients were excluded if they underwent extracorporeal life support or were on mechanical ventilation for less than one day. The primary outcome was 28-day mortality. Bayesian joint models were employed to estimate the strength of associations over time.

**Results:**

A total of 2,851 patients were included in our analysis. The overall 28-day mortality rate was 21.3%, with a median duration of invasive mechanical ventilation of 9 days (IQR: 4–28 days). After adjustment, each daily increment in PaCO_2_ (HR 1.008, 95% CI 0.997–1.018) was not associated with mortality, while a daily increment in VR (HR 1.548, 95% CI 1.309–1.835) was associated with increased mortality. This association persisted during the prolonged stages (Days 0–23) of mechanical ventilation. Furthermore, a significant increase in the risk of death was related to daily exposure to VR > 2 (HR 1.088 per day, 95% CI 1.034–1.147) and its cumulative effect (HR 1.085 per area, 95% CI 1.050–1.122), whereas PaCO_2_ was found to be insignificant.

**Conclusion:**

VR, which reflects ventilatory inefficiency, should be closely monitored during invasive mechanical ventilation. Cumulative exposure to high intensities of VR may be associated with increased mortality in patients with ARDS.

**Supplementary Information:**

The online version contains supplementary material available at 10.1186/s13613-025-01427-1.

## Background

Acute respiratory distress syndrome (ARDS) is a life-threatening respiratory failure characterized by hypoxemia, and impaired clearance of carbon dioxide (CO_2_), with most patients requiring invasive mechanical ventilation for support [1, 2]. The primary goals of mechanical ventilation in ARDS management are to maintain adequate oxygenation and ventilation [3, 4]. Clinically, oxygenation is typically assessed by the ratio of arterial oxygen partial pressure to inspired fraction of oxygen (PaO_2_/FiO_2_) [1], while ventilation is primarily reflected by arterial carbon dioxide pressure (PaCO_2_), which depends on both CO_2_ production and alveolar ventilation. Despite its significance, the relationship between prolonged hypercarbia and prognosis in ARDS patients remains unresolved, with no consensus currently established [5, 6].

Ventilatory inefficiency, however, can manifest in clinical practice through increased PaCO_2_, elevated minute ventilation, or a combination of both. Therefore, a composite index, the ventilatory ratio (VR), which compares actual measurements of minute ventilation and PaCO_2_ to predicted values, has been proposed to better reflect ventilatory inefficiency [7]. Despite the potential of VR as a marker, previous studies investigating its association with ARDS prognosis have reported inconsistent results, likely due to limitations in sample sizes and the variability in controlling for confounding factors [8, 9]. Furthermore, findings from studies focusing on COVID-related ARDS may not be generalizable to the broader population of patients with classical ARDS due to differences in lung volume, respiratory mechanics, and ventilatory requirements [10–12]. Additionally, earlier researches on PaCO_2_ and VR often focused on single measurements upon admission or was restricted to the early phases of ARDS [5, 6, 12–14]. As a result, it remains unclear whether the relationship between ventilatory inefficiency and mortality in ARDS patients persists over time.

In this study, our primary objective was to evaluate the impact of time-varying exposure to ventilatory inefficiency—measured by PaCO_2_ and VR—on 28-day mortality in patients with ARDS. We also sought to determine whether the strength of these associations persisted over time and to quantify the cumulative impact of these exposures over the study period.

## Methods

### Study design and patients

We performed a secondary analysis of data from four published randomized controlled trials: FACTT (Fluid and Catheter Treatment Trial) [15, 16], ALTA (Albuterol for the Treatment of Acute Lung Injury) [17], EDEN (Early vs Delayed Enteral Nutrition in ARDS) [18], and SAILS (Statins for Acutely Injured Lungs) [19]. These trials focused on patients with classical ARDS and adhered to the low tidal volume ventilation protocol, reflecting current clinical practice standards. The details of these trials are summarized in Additional file 1: Table S1.

Included patients were adult subjects who were intubated and received mechanical ventilation. Exclusion criteria were: (1) Patients under the age of 18, (2) Patients lacking measurements of predicted body weight (PBW) or height needed to calculate VR, (3) Patients treated with extracorporeal life support, (4) Patients receiving invasive ventilation for less than one day, and (5) Patients lacking on-study ventilatory parameter measurements.

All studies obtained informed consent from patients and were approved by the respective institutional review boards. This study protocol, titled “The Association Between Time-Varying Intensity of Ventilatory Indices and Mortality in Patients with Acute Respiratory Distress Syndrome,” was approved by the Research Ethics Commission of Zhongda Hospital, Southeast University (Approval ID: 2023ZDSYLL158-P01, 20,230,511). The commission determined that the activities involved in this research did not constitute human subjects research, thus waiving the requirement for informed consent. Data were obtained from the Biologic Specimen and Data Repository Information Coordinating Center (BioLINCC, https://biolincc.nhlbi.nih.gov). This article complies with the STROBE (Strengthening the Reporting of Observational Studies in Epidemiology) guidelines for observational studies.

### Data collection

In the ARDS Network trials, Day 0 was defined as the day of randomization following patient enrollment. Per trial protocol, patients were required to begin low tidal volume ventilation within one hour of meeting the inclusion criteria. Demographic data collected included age, gender, primary risk factors for ARDS, chronic comorbidities, and severity of illness as measured by the Acute Physiology and Chronic Health Evaluation (APACHE) III score at admission.

Longitudinal data were collected for preselected variables, which were categorized under “on-study ventilator parameters.” These included arterial blood gases and ventilator parameters, which were recorded as close as possible to 08:00 AM on Days 0–4, 7, 12/14, 21, and 28 of mechanical ventilation. Ventilatory ratio is defined as [minute ventilation(mL/min) × PaCO_2_(mmHg)]/[PBW(kg) × 100(mL/min) × 37.5(mmHg)]. Data collection spanned from Day 0 until the earliest of the following events: death, discharge from the intensive care unit (ICU), liberation from mechanical ventilation for more than 48 h, or 28 days of ICU stay. The primary outcome of interest was 28-day mortality.

### Statistical analysis

Categorical variables were summarized as proportions, while continuous variables were presented as means (standard deviations) or medians [interquartile ranges (IQRs)], depending on their distribution. Comparisons between groups were conducted using the Student’s t-test or Mann–Whitney U test for continuous variables, and the Chi-squared (Χ^2^) test or Fisher’s exact test for categorical variables, as appropriate.

We first employed cause-specific Cox proportional hazard models to evaluate the relationship between baseline ventilatory parameters (PaCO_2_ and VR) and 28-day mortality. Restricted cubic splines were used within these models to express hazard ratios (HRs) and corresponding two-sided 95% credible intervals (CIs). Based on the model outputs, we identified approximate thresholds for PaCO_2_ and VR.

Potential confounders were identified and assessed based on directed acyclic graphs (DAG) and change-in-estimate methods, while addressing collinearity and handling missing data to ensure robust and unbiased variable selection. (Additional file 1: Fig. S1-S2 & Table S2). The following covariates were selected for inclusion in the models:

- Baseline confounders: Age, gender, body mass index (BMI), APACHE III score, comorbidities (Hypertension, Diabetes Mellitus, Chronic Pulmonary Disease), tidal volume per predicted body weight (PBW), positive end-expiratory pressure (PEEP), use of sedatives or neuromuscular blocking agents (NMBAs) (Yes/No), and use of vasopressors or inotropes(Yes/No).
- Time-varying confounder: PaO_2_/FiO_2_.

To assess the association between longitudinal ventilatory parameters (PaCO_2_ and VR) and mortality, we utilized Bayesian joint models incorporating shared random effects. These models allow for the analysis of correlations between individual longitudinal profiles and 28-day mortality by accounting for non-random dropouts during follow-up. Shared parameter joint models combine mixed-effects models with Cox regression, linking patient-specific longitudinal trajectories to their prognosis. This approach assumes that latent random effects fully account for correlations between longitudinal exposures and outcomes after adjusting for covariates [20, 21].

Natural cubic splines were applied within both fixed-effects and random-effects models to accommodate any nonlinearity in the longitudinal exposure profiles. Estimations were performed using the JMbayes2 package, with additional details provided in Additional file 1 [22]. Standard Markov Chain Monte Carlo diagnostics were employed to assess model convergence [23], and the results were derived from the posterior distribution to predict the hazard of death within 28 days.

Given that the initial joint model assumed a constant strength of association over time, we incorporated an interaction term with a natural cubic spline of time to assess whether the association between ventilatory inefficiency and mortality persisted across time. We also explored the time-varying impact of daily high exposure to ventilatory inefficiency, defined as PaCO_2_ > 50 mmHg or VR > 2, on 28-day mortality. Additionally, we quantified the effect of cumulative exposure to elevated PaCO_2_ or VR by calculating the area beneath each subject’s longitudinal profile and above the specified thresholds, across the corresponding time period.

To mitigate bias resulting from missing data, we employed Multiple Imputation by Chained Equations (MICE), generating five imputed datasets to address the missing values. To verify the robustness of our findings, we repeated the joint model analyses using complete cases without imputation. We also performed subgroup analyses based on several factors, including age, gender, APACHE score, PaO_2_/FiO_2_ ratio, primary ARDS risk factors, and the use of sedatives or neuromuscular blocking agents (NMBAs), vasopressors or inotropes. For continuous variables, cut-off points were derived from existing knowledge or the interquartile ranges (IQRs). A P value < 0.05 (two-tailed) was considered statistically significant. All statistical analyses were conducted using R version 4.1.2 (R Core Team 2021, Vienna, Austria).

## Results

### Patients in the study

After reviewing data from 3027 patients, we included 2851 patients in the final analysis (Additional file 1: Fig. S3). The median age of the patients was 52 years (IQR 40–63), and 51.6% were male. The primary etiologies of ARDS were pneumonia (58.1%), sepsis (19.9%), and aspiration (11.4%). The median duration of invasive mechanical ventilation was 9 days (IQR 4–28 days), and the overall 28-day mortality rate was 21.3%.

Compared with survivors, non-survivors were older, had lower BMI, higher APACHE III scores, and were less likely to have received sedatives or NMBAs. In the cohort, tidal volumes were ≤ 8 mL/kg PBW in 1886 patients (79.0%), accounting for missing values (Additional file 1: Table S2). On Day 0 of mechanical ventilation, plateau pressures ≤ 30 cmH_2_O and driving pressures ≤ 15 cmH_2_O were observed in 1,542 patients (80.2%) and 952 patients (49.6%), respectively. Importantly, there were no significant differences in peak inspiratory pressure or driving pressure between survivors and non-survivors (Table 1).

**Table 1:** Baseline characteristics at Day 0 of mechanical ventilation

Characteristics	All patients (n = 2851)	Survivors (n = 2243)	Non-survivors (n = 608)	P value
Age, years	52 [40,63]	50 [40,60]	58 [46,71]	< 0.001
Male, no (%)	1470 (51.6)	1136 (50.6)	334 (55.0)	0.060
BMI	28.1 [23.6,33.8]	28.5 [23.8,34.0]	27.0 [22.8,33.0]	0.002
APACHE III score	91 [72,112]	86 [69,106]	108 [91,126]	< 0.001
Primary risk factor of ARDS—no (%)				
Trauma	135 (4.7)	122 (5.4)	13 (2.1)	0.001
Sepsis	568 (19.9)	426 (19.0)	142 (23.4)	0.018
Multiple Transfusion	33 (1.2)	28 (1.2)	5 (0.8)	0.514
Aspiration	324 (11.4)	272 (12.1)	52 (8.6)	0.018
Pneumonia	1656 (58.1)	1287 (57.4)	369 (60.8)	0.140
Others	142 (5.0)	116 (5.2)	26 (4.3)	0.432
Comorbidities—no (%)				
Solid tumor with metastasis	66 (2.3)	38 (1.7)	28 (4.6)	< 0.001
Immune suppression	311 (11.0)	199 (9.0)	112 (18.6)	< 0.001
Hepatic failure	30 (1.1)	14 (0.6)	16 (2.7)	< 0.001
Cirrhosis	128 (4.5)	81 (3.7)	47 (7.8)	< 0.001
Diabetes mellitus	646 (22.9)	510 (23.0)	136 (22.6)	0.865
Hypertension	1105 (41.3)	854 (40.5)	251 (44.2)	0.121
Congestive heart failure	142 (5.3)	110 (5.2)	32 (5.6)	0.765
Chronic pulmonary disease	317 (11.8)	232 (11.0)	85 (15.0)	0.011
Ventilator settings				
FiO_2_, %	0.6 [0.4,0.7]	0.5 [0.4,0.7]	0.6 [0.5,0.8]	< 0.001
Set respiratory rate, min^−1^	20 [16, 28]	20 [16, 26]	22 [16, 28]	0.029
Total respiratory rate, min^−1^	25 [20, 30]	24 [20, 30]	26 [21, 31]	< 0.001
Total minute ventilation, L/min	11.0 [8.9,13.5]	10.8 [8.8,13.3]	11.6 [9.4,14.2]	< 0.001
Tidal volume, mL/kg PBW	6.6 [6.0,7.7]	6.6 [6.0,7.7]	6.6 [6.0,7.8]	0.822
PEEP, cmH_2_O	10 [5, 12]	10 [5, 12]	10 [5, 12]	0.097
Plateau Pressure, cmH_2_O	24 [20, 28]	24 [20, 28]	25 [20, 29]	0.032
Peak Inspiratory Pressure, cmH_2_O	29 [23,35]	29 [23,34]	29 [24,36]	0.103
Driving pressure, cmH_2_O	15 [11, 18]	14 [11, 18]	15 [11, 20]	0.102
Compliance, ml/cmH_2_O	29.4 [22.4,38.5]	29.7 [22.8,38.5]	27.8 [20.8,37.6]	0.043
Gas exchange				
Arterial pH	7.37 [7.31,7.42]	7.37 [7.31,7.42]	7.36 [7.28,7.41]	< 0.001
SpO_2_, %	95 [80,98]	95 [80,98]	94 [89,98]	0.3147
PaO_2_, mmHg	82 [68,104]	82 [69,104]	79 [66,103]	0.035
PaCO_2_, mmHg	39 [34,45]	39 [34,45]	39 [33,45]	0.564
PaO_2_/FiO_2_, mmHg	151 [107,210]	154 [112,212]	138 [94,200]	< 0.001
Ventilatory ratio	1.8 [1.5,2.3]	1.8 [1.4,2.2]	1.9 [1.5,2.5]	< 0.001
Intervention				
Fluid balance, mL	1950 [458,3996]	1845 [409,3907]	2331 [824,4587]	< 0.001
Sedatives or NMBAs use, no (%)	2548 (89.5)	2033 (90.6)	515 (85.1)	< 0.001
Vasopressors or inotropes use, no (%)	1288 (45.2)	947 (42.2)	341 (56.2)	< 0.001

Data are expressed as n (%) and median (interquartile range)

*BMI* body mass index, *APACHE* Acute Physiology and Chronic Health Evaluation, *ARDS* acute respiratory distress syndrome, *FiO*_*2*_ inspired fraction of oxygen, *PBW* predicted body weight, *PEEP* positive end-expiratory pressure, *pH* potential of hydrogen, *SpO*_*2*_ peripheral capillary oxygen saturation, *PaO*_*2*_ partial pressure of oxygen, *PaCO*_*2*_ arterial carbon dioxide pressure, *PaO*_*2*_*/FiO*_*2*_ ratio of arterial oxygen partial pressure to inspired fraction of oxygen, *NMBAs* neuromuscular blocking agents

### Association of mortality with ventilatory parameters at baseline

**Fig. 1:**
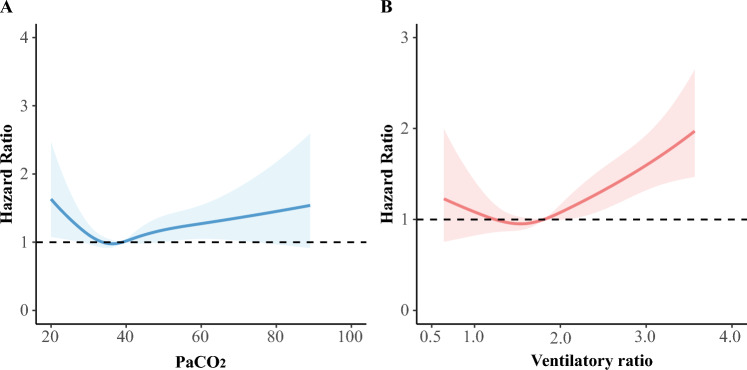
Outcomes in relation to ventilatory parameters at baseline. **A** Adjusted hazard ratio for 28-day mortality predicted by baseline PaCO_2_ using a cause-specific Cox proportional hazard model. **B** Adjusted hazard ratio for 28-day mortality predicted by baseline ventilatory ratio using a cause-specific Cox proportional hazard model. *PaCO*_*2*_ arterial carbon dioxide pressure

A U-shaped relationship was identified between PaCO_2_ or VR and the risk of 28-day mortality. Inefficient ventilation, indicated by hypercapnia (PaCO_2_ > 50 mmHg) or high VR (VR > 2), was associated with an increased hazard of death within 28 days after adjustment (Fig. 1A and Fig. 1B). After adjusting for multiple covariates, including age, gender, BMI, APACHE III score, comorbidities (hypertension, diabetes, chronic pulmonary disease), tidal volume per PBW, PEEP, use of sedatives or NMBAs, use of vasopressors or inotropes, and PaO_2_/FiO_2_, only the ventilatory ratio (VR) at baseline remained significantly associated with mortality (HR 1.101, 95% CI 1.009–1.201). PaCO_2_, on the other hand, was not significantly associated with mortality after adjustment (HR 1.003, 95% CI 0.995–1.011) (Additional file 1: Table S3).

Of the total cohort, 2769 patients (97.1%) had complete PaCO_2_ data on Day 0, with 12.4% of patients experiencing impaired carbon dioxide clearance (PaCO_2_ > 50 mmHg). Additionally, 2673 patients (93.8%) had complete VR data on Day 0, and 38.4% of patients had a VR > 2. Significant variations in patient characteristics and interventions were noted based on PaCO_2_ and VR levels at baseline (Additional file 1: Tables S4-S5).

### Effect of time-varying intensities of ventilatory parameters

In the joint model analysis, time-varying ventilatory ratio (VR) (HR 1.548, 95% CI 1.309–1.835) was significantly associated with an increased risk of death after adjusting for covariates. In contrast, time-varying PaCO_2_ (HR 1.008, 95% CI 0.997–1.018) showed no significant association with mortality, consistent with the results from the baseline cause-specific Cox proportional hazard model (Table 2).

**Table 2:** The effect of time-varying intensities of ventilatory parameters on mortality

	Model with PaCO_2_		Model with VR	
	HR (95% CI)	P value	HR (95% CI)	P value
Baseline characteristics				
Age, years	1.023(1.016–1.031)	< 0.001	1.023(1.016–1.031)	< 0.001
Gender	1.097(0.926–1.296)	0.268	1.112(0.935–1.312)	0.205
BMI	1.000(0.989–1.010)	0.922	1.000(0.989–1.010)	0.929
APACHE III	1.020(1.017–1.023)	< 0.001	1.020(1.016–1.023)	< 0.001
Hypertension	0.867(0.716–1.047)	0.138	0.868(0.723–1.042)	0.137
Diabetes mellitus	0.832(0.684–1.016)	0.069	0.835(0.693–1.021)	0.078
Chronic pulmonary disease	1.212(0.976–1.493)	0.085	1.213(0.971–1.507)	0.092
Tidal volume, mL/kg PBW	0.997(0.966–1.015)	0.965	0.994(0.960–1.014)	0.805
Positive end-expiratory pressure	1.021(0.998–1.044)	0.067	1.019(0.996–1.043)	0.107
Use of sedatives or NMBAs	0.595(0.480–0.753)	< 0.001	0.594(0.476–0.747)	< 0.001
Use of vasopressors or inotropes	1.027(0.859–1.226)	0.779	1.053(0.882–1.259)	0.569
Time-varying variables				
PaO_2_/FiO_2_	0.998(0.997–1.000)	0.058	1.000 (0.998–1.002)	0.107
PaCO_2_	1.008(0.997–1.018)	0.144		
Ventilatory ratio			1.548(1.309–1.835)	< 0.001

Number of subjects: 2851; Number of events: 608 (21.3%); Number of observations: 15,989. The hazard ratios were the adjusted hazard ratios associated with 1 increment in the given variable

*PaCO*_*2*_ arterial carbon dioxide pressure, *VR* ventilatory ratio, *HR* hazard ratio, *CI* confidence interval, *BMI* body mass index, *APACHE* Acute Physiology and Chronic Health Evaluation, *PBW* predicted body weight, *NMBAs* neuromuscular blocking agents, *PaO*_*2*_*/FiO*_*2*_ ratio of arterial oxygen partial pressure to inspired fraction of oxygen

**Fig. 2:**
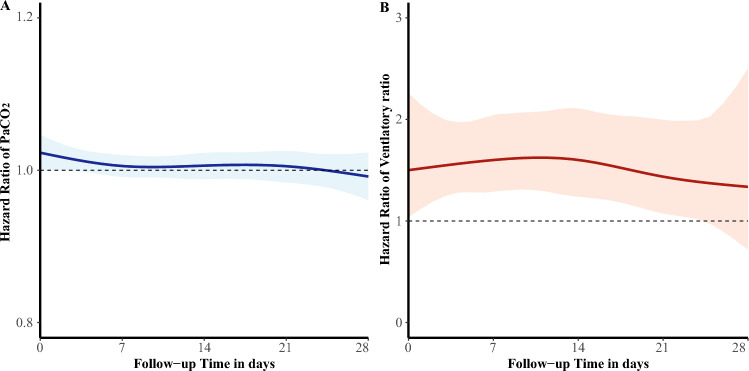
Time-varying effect of ventilatory parameters and mortality. **A** Adjusted time-varying effect of PaCO_2_ and 28-day mortality using Bayesian joint models. **B** Adjusted time-varying effect of ventilatory ratio and 28-day mortality using Bayesian joint models. *PaCO*_*2*_ arterial carbon dioxide pressure

The lack of a significant correlation between PaCO_2_ intensity and mortality persisted throughout the entire ventilation period, as illustrated in Fig. 2A. By contrast, VR was consistently associated with 28-day mortality over the course of mechanical ventilation, as shown in Fig. 2B. Traceplots and density plots confirmed good convergence of the joint models (Additional file 1: Fig. S4-S5).

### Cumulative effect of exposure to high intensities of ventilatory inefficiency

The risk of mortality increased consistently with daily exposure to PaCO_2_ > 50 mmHg and VR > 2. Each additional day of exposure to elevated VR (> 2) was associated with a heightened risk of death after adjustment (HR 1.088 per day, 95% CI 1.034–1.147). However, prolonged exposure to high PaCO_2_ levels (> 50 mmHg) did not show a significant effect on mortality (HR 1.040 per day, 95% CI 0.979–1.104) (Table 3).

**Table 3:** The effect of time-varying exposure to hypercapnia or high VR on mortality

	Model with PaCO_2_		Model with VR	
	HR (95% CI)	P value	HR (95% CI)	P value
Baseline characteristics				
Age, years	1.023(1.016–1.031)	< 0.001	1.023(1.016–1.031)	< 0.001
Gender	1.101(0.928–1.305)	0.267	1.101(0.927–1.306)	0.285
BMI	1.000(0.989–1.010)	0.883	1.000(0.989–1.010)	0.856
APACHE III	1.020(1.017–1.023)	< 0.001	1.019(1.016–1.022)	< 0.001
Hypertension	0.867(0.720–1.043)	0.127	0.874(0.729–1.051)	0.152
Diabetes mellitus	0.831(0.679–1.013)	0.068	0.833(0.685–1.008)	0.060
Chronic pulmonary disease	1.211(0.962–1.518)	0.105	1.220(0.974–1.538)	0.076
Tidal volume, mL/kg PBW	0.998(0.967–1.015)	0.995	0.995(0.964–1.014)	0.853
Positive end-expiratory pressure	1.022(0.999–1.045)	0.062	1.021 (0.998–1.045)	0.072
Use of sedatives or NMBAs	0.598(0.477–0.756)	< 0.001	0.595(0.479–0.741)	< 0.001
Use of vasopressors or inotropes	1.027 (0.863–1.225)	0.761	1.049 (0.889–1.260)	0.587
Time-varying variables				
PaO_2_/FiO_2_	0.999(0.997–1.000)	0.110	0.999 (0.997–1.001)	0.511
Any exposure to hypercapnia (PaCO_2_ > 50 mmHg)	1.040(0.979–1.104)	0.203		
Any exposure to high VR (Ventilatory ratio > 2)			1.088(1.034–1.147)	0.002

Number of subjects: 2851; Number of events: 608 (21.3%); Number of observations: 15,989. The hazard ratios were the adjusted hazard ratios comparing higher versus lower levels of the variable

*VR* ventilatory ratio, *HR* hazard ratio, *CI* confidence interval, *BMI* body mass index, *APACHE* Acute Physiology and Chronic Health Evaluation, *PBW* predicted body weight, *NMBAs* neuromuscular blocking agents, *PaO*_*2*_*/FiO*_*2*_ ratio of arterial oxygen partial pressure to inspired fraction of oxygen, *PaCO*_*2*_ arterial carbon dioxide pressure

Additionally, a cumulative impact of higher intensities of ventilatory inefficiency was observed. An increased risk of mortality was linked to a larger cumulative area of VR > 2 (HR 1.085 per area, 95% CI 1.050–1.122) after adjustments. Conversely, cumulative exposure to PaCO_2_ > 50 mmHg did not significantly influence 28-day mortality (HR 1.002 per area, 95% CI 0.999–1.004) (Additional file 1: Table S6).

### Subgroup analyses and sensitivity analyses

**Fig. 3:**
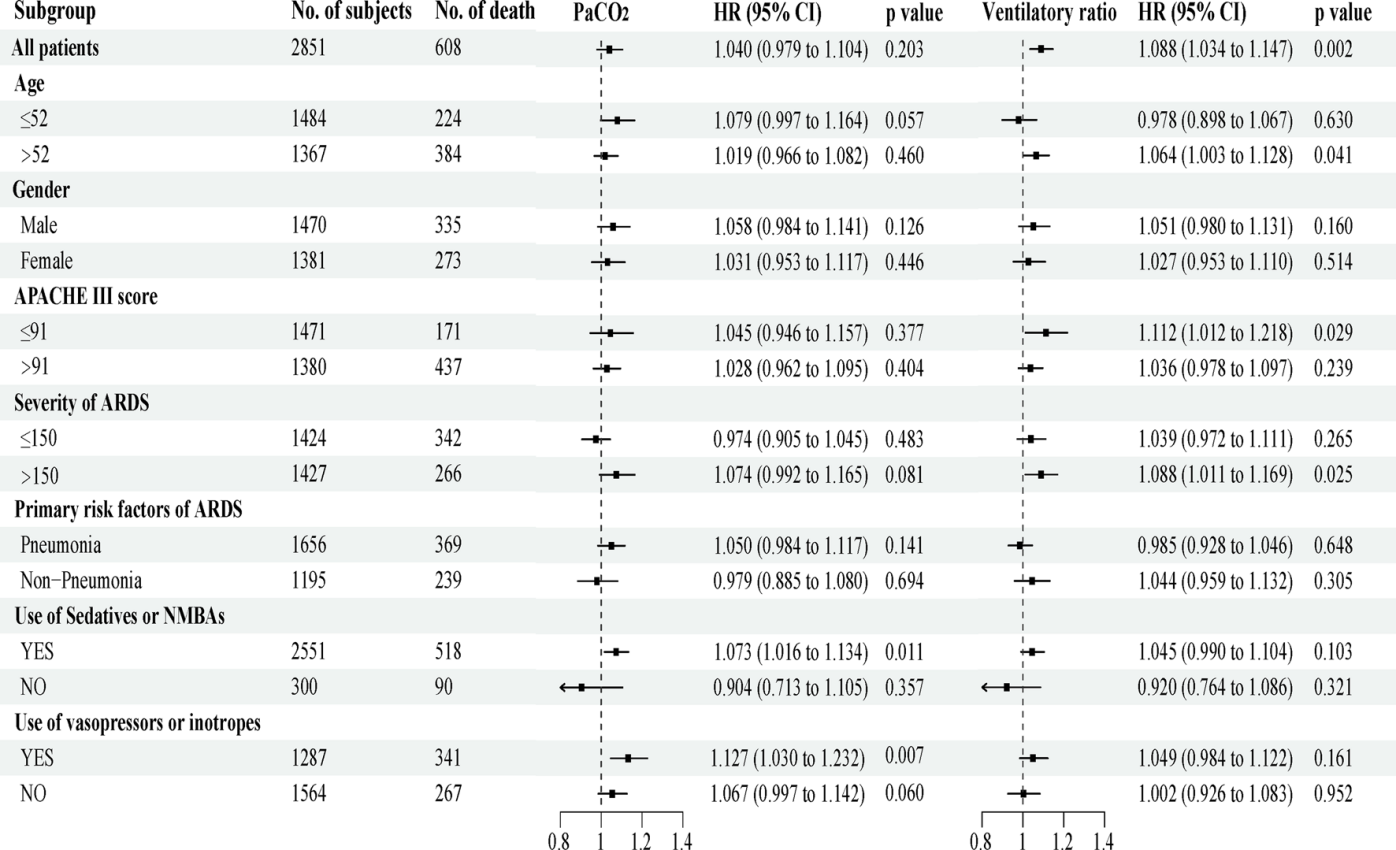
Subgroup analyses of the association between any exposure to hypercapnia or high VR. The hazard ratios were the adjusted hazard ratios comparing higher versus lower levels of the variable. *PaCO*_*2*_ arterial carbon dioxide pressure, *HR* hazard ratio, *CI* confidence interval, *APACHE* Acute Physiology and Chronic Health Evaluation, *PaO*_*2*_*/FiO*_*2*_ ratio of arterial oxygen partial pressure to inspired fraction of oxygen, *NMBAs* neuromuscular blocking agents

Subgroup analyses explored the relationship between time-varying hypercapnia and high VR with 28-day mortality (Fig. 3). The association between high VR and mortality appeared to be more pronounced in patients with lower severity of illness, as indicated by an APACHE III score ≤ 91 (HR 1.112 per day, 95% CI 1.012–1.218) or PaO_2_/FiO_2_ > 150 (HR 1.088 per day, 95% CI 1.011–1.169). Regarding hypercapnia, the hazard of death was elevated in patients receiving sedatives or neuromuscular blocking agents (NMBAs) (HR 1.073 per day, 95% CI 1.016–1.134) and in those receiving vasopressors or inotropes (HR 1.127 per day, 95% CI 1.030–1.232).

To test the robustness of our initial findings, we repeated the joint model analyses after excluding patients with missing longitudinal data on PaCO_2_ and VR during follow-up. The results from the complete datasets were consistent with those derived from imputed datasets (PaCO_2_: HR 1.073, 95% CI 0.998–1.158 vs. VR: HR 1.088, 95% CI 1.021–1.164), confirming the reliability of our findings (Additional file 1: Table S7).

## Discussion

In clinical practice, ventilation assessment has often been overshadowed by the emphasis on oxygenation during mechanical ventilation in patients with ARDS. Indices such as PaCO_2_ and the ventilatory ratio (VR), which are readily available and indicative of ventilatory inefficiency, have not been fully appreciated and require more attention in clinical studies [24]. Our study highlights the longitudinal relationship between PaCO_2_, VR, and 28-day mortality, suggesting that clinicians should attach importance to VR throughout the period of mechanical ventilation. We also found that time- and dose-dependent exposure to high levels of ventilatory inefficiency, as measured by VR > 2, rather than PaCO_2_ > 50 mmHg, was associated with increased mortality in ARDS.

PaCO_2_, a frequently monitored parameter linked to alveolar ventilation, can rise due to ventilatory inefficiency or hypoventilation. Ventilatory inefficiency indicated by hypercapnia showed no significant association with ARDS prognosis when compared to VR. A possible explanation for this discrepancy is the beneficial effects of permissive hypercapnia in patients undergoing lung-protective ventilation [25]. By tolerating elevated PaCO_2_ levels, permissive hypercapnia mitigates acute lung injury through reduced mechanical stretch and anti-inflammatory effects [26, 27]. Moreover, hypercapnia improves ventilation-perfusion matching by enhancing bronchodilation and hypoxic vasoconstriction, contributing to better pulmonary and systemic oxygenation [28].

The dimensionless VR is widely recognized as a marker for assessing pulmonary dead space [29]. Increased pulmonary dead space reflects the lungs' inefficiency in eliminating CO_2_, which can lead to hypercapnia. Given that the anatomical portion of dead space is generally constant, increasing tidal volumes (VT) with a stable respiratory rate would increase alveolar ventilation, effectively lowering PaCO_2_. This tradeoff is captured by VR [30]. Ventilatory inefficiency, reflected by high VR(VR > 2) is independently associated with increased case fatality in patients with ARDS [31]. The utility of VR for quantifying physiological dead space, however, may be limited by factors such as venous admixture and the CO_2_ production [9]. As such, VR serves as a measure of global gas exchange efficiency and allows the quantification of ventilation-perfusion mismatch [29]. VR shows promise as a simple method to stratify ARDS patients that is not captured by conventional oxygenation indices.

We further attempted to address ARDS heterogeneity through subgroup analyses. The stronger association between higher VR and mortality in less severe cases, as indicated by APACHE III and PaO_2_/FiO_2_ scores, appears exploratory in nature, consistent with previous study [32]. A higher VR in these cases may reflect an early maladaptive response to underlying pathophysiological processes, such as subtle respiratory muscle fatigue or early ventilation-perfusion mismatch. In contrast, in patients with more severe disease, elevated VR may simply be a marker of overall critical illness, where multiple organ systems are already compromised [33]. This could dilute the specific impact of VR on mortality. Future prospective and interventional studies, guided by focused hypotheses and mechanistic insights, are needed to better understand the clinical significance of VR across varying severities of ARDS.

Our study has important implications for interpreting previous research. First, we extended the assessment period between VR and its association with mortality, capturing its dynamic evolution over the course of the ventilation. Second, Bayesian joint models were employed in our study to account for both baseline and time-varying confounders, offering a more precise estimation of temporal associations. Third, we identified clinically meaningful thresholds for VR > 2—aligning with evidence from previous studies [8, 31]. Finally, the larger sample size in our study enhances the statistical power, improving the robustness and external validity of the observed relationships. Overall, our results emphasize the role of VR in prognostication and highlight the importance of adequately powered studies in this area.

Several limitations of our study should be acknowledged. First, the study is a retrospective observational analysis based on datasets from four randomized controlled trials. This design introduces inevitable challenges, such as missing values and the inability to infer causality. Second, our dataset consists of arterial blood gas and ventilator data collected at a standardized time each morning. It is possible that these measurements were not taken simultaneously or did not fully capture the ventilatory parameters throughout the day. To address these limitations, a prospective study is warranted to confirm these findings and explore their potential implications for clinical interventions.

## Conclusion

In conclusion, ventilatory inefficiency, as reflected by VR, is associated with increased mortality in patients with ARDS undergoing invasive mechanical ventilation. Long-term exposure to high VR levels correlates with higher mortality, underscoring the importance of vigilant monitoring. However, given the retrospective design, these findings suggest an association rather than causation and warrant further validation through well-designed prospective studies.

## Supplementary Information

Additional file 1:A. Additional Methods: 1. Data collection. 2. Approach to missing data. 3. Bayesian joint model. B. Additional Results: Table S1 Characteristics of enrolled clinical trials. Table S2 Percentage of missing data in the baseline and dynamic cohort. Table S3 Adjusted hazard ratios of cause-specific Cox proportional model at baseline. Table S4 Baseline characteristics of patients stratified by PaCO_2_. Table S5 Baseline characteristics of patients stratified by ventilatory ratio. Table S6 The effect of cumulative exposure to hypercapnia or high VR on mortality. Table S7 The effect of time-varying exposure to hypercapnia or high VR after excluding missing data. Fig. S1 Directed acyclic graph illustrating the potential confounders on the association of ventilatory parameters and mortality. Fig. S2 Correlation matrix for baseline (A) and dynamic (B) covariates. Fig. S3 Study flow diagram in the present study. Fig. S4 Diagnostic plot of the joint model based on PaCO_2_. Fig. S5 Diagnostic plot of the joint model based on ventilatory ratio

## Data Availability

The datasets supporting the conclusions of this article are available from the Biologic Specimen and Data Repository Information Coordinating Center of the National Heart, Lung, and Blood Institute (NHLBI) and can be accessed at https://biolincc.nhlbi.nih.gov/home/.

## Abbreviations

ALTA: Albuterol for the Treatment of Acute Lung Injury
APACHE: Acute Physiology and Chronic Health Evaluation
ARDS: Acute respiratory distress syndrome
BMI: Body mass index
CI: Credible intervals
CO_2_: Carbon dioxide
COVID: Coronavirus disease
DAG: Directed acyclic graph
EDEN: Early vs Delayed Enteral Nutrition in ARDS
FACTT: Fluid and Catheter Treatment Trial
HR: Hazard ratios
ICU: Intensive care unit
IQR: Interquartile ranges
MICE: Multiple Imputation by Chained Equations
NMBAs: Neuromuscular blocking agents
PaCO_2_: Arterial carbon dioxide pressure
PaO_2_/FiO_2_: Arterial oxygen partial pressure to inspired fraction of oxygen
PBW: Predicted body weight
PEEP: Positive end-expiratory pressure
SAILS: Statins for Acutely Injured Lungs
VR: Ventilatory ratio

## References

[1] Definition Task Force ARDS, Ranieri VM, Rubenfeld GD, Thompson BT, Ferguson ND, Caldwell E, et al. Acute respiratory distress syndrome: the Berlin definition. JAMA. 2012;307(23):2526–33. 22797452 10.1001/jama.2012.5669

[2] Fan E, Del Sorbo L, Goligher EC, Hodgson CL, Munshi L, Walkey AJ, et al. An official American thoracic society/European society of intensive care medicine/society of critical care medicine clinical practice guideline: mechanical ventilation in adult patients with acute respiratory distress syndrome. Am J Respir Crit Care Med. 2017;195(9):1253–63. 28459336 10.1164/rccm.201703-0548ST

[3] Radermacher P, Maggiore SM, Mercat A. Fifty years of research in ARDS. Gas exchange in acute respiratory distress syndrome. Am J Respir Crit Care Med. 2017;196(8):964–84. 28406724 10.1164/rccm.201610-2156SO

[4] Pelosi P, Ball L, Barbas CSV, Bellomo R, Burns KEA, Einav S, et al. Personalized mechanical ventilation in acute respiratory distress syndrome. Crit Care. 2021;25(1):250. 34271958 10.1186/s13054-021-03686-3 PMC8284184

[5] Madotto F, Rezoagli E, McNicholas BA, Pham T, Slutsky AS, Bellani G, SAFE Investigators and the ESICM Trials Group, et al. LUNG Patterns and impact of arterial CO(2) management in patients with acute respiratory distress syndrome: insights from the LUNG SAFE study. Chest. 2020;158(5):1967–82. 32589951 10.1016/j.chest.2020.05.605

[6] Nin N, Muriel A, Peñuelas O, Brochard L, Lorente JA, Ferguson ND, VENTILA Group, et al. Severe hypercapnia and outcome of mechanically ventilated patients with moderate or severe acute respiratory distress syndrome. Intensive Care Med. 2017;43(2):200–8. 28108768 10.1007/s00134-016-4611-1 PMC5630225

[7] Sinha P, Fauvel NJ, Singh S, Soni N. Ventilatory ratio: a simple bedside measure of ventilation. Br J Anaesth. 2009;102(5):692–7. 19346233 10.1093/bja/aep054

[8] Sinha P, Calfee CS, Beitler JR, Soni N, Ho K, Matthay MA, et al. Physiologic analysis and clinical performance of the ventilatory ratio in acute respiratory distress syndrome. Am J Respir Crit Care Med. 2019;199(3):333–41. 30211618 10.1164/rccm.201804-0692OC PMC6363976

[9] Maj R, Palermo P, Gattarello S, Brusatori S, D’Albo R, Zinnato C, et al. Ventilatory ratio, dead space, and venous admixture in patients with acute respiratory distress syndrome. Br J Anaesth. 2023;130(3):360–7. 36470747 10.1016/j.bja.2022.10.035 PMC9718027

[10] Pozzi T, Collino F, Brusatori S, Romitti F, Busana M, Moerer O, et al. Specific respiratory system compliance in COVID-19 and non-COVID-19 acute respiratory distress syndrome. Am J Respir Crit Care Med. 2023;208(3):328–30. 37311259 10.1164/rccm.202302-0223LE PMC10395712

[11] Liu X, Liu X, Xu Y, Xu Z, Huang Y, Chen S, et al. Ventilatory ratio in hypercapnic mechanically ventilated patients with COVID-19-associated acute respiratory distress syndrome. Am J Respir Crit Care Med. 2020;201(10):1297–9. 32203672 10.1164/rccm.202002-0373LE PMC7233337

[12] Beloncle F, Studer A, Seegers V, Richard JC, Desprez C, Fage N, et al. Longitudinal changes in compliance, oxygenation and ventilatory ratio in COVID-19 versus non-COVID-19 pulmonary acute respiratory distress syndrome. Crit Care. 2021;25(1):248. 34266454 10.1186/s13054-021-03665-8 PMC8280689

[13] Morales-Quinteros L, Neto AS, Artigas A, Blanch L, Botta M, Kaufman DA, PRoVENT-COVID Study Group, et al. Dead space estimates may not be independently associated with 28-day mortality in COVID-19 ARDS. Crit Care. 2021;25(1):171. 34001222 10.1186/s13054-021-03570-0 PMC8127435

[14] Torres A, Motos A, Riera J, Fernández-Barat L, Ceccato A, Pérez-Arnal R, CIBERESUCICOVID Project (COV20/00110, ISCIII), et al. The evolution of the ventilatory ratio is a prognostic factor in mechanically ventilated COVID-19 ARDS patients. Crit Care. 2021;25(1):331. 34517881 10.1186/s13054-021-03727-x PMC8436582

[15] National Heart, Lung, and Blood Institute Acute Respiratory Distress Syndrome (ARDS) Clinical Trials Network, Wheeler AP, Bernard GR, Thompson BT, Schoenfeld D, Wiedemann HP, de Boisblanc B, et al. Pulmonary-artery versus central venous catheter to guide treatment of acute lung injury. N Engl J Med. 2006;354(21):2213–24. 16714768 10.1056/NEJMoa061895

[16] National Heart, Lung, and Blood Institute Acute Respiratory Distress Syndrome (ARDS) Clinical Trials Network, Wiedemann HP, Wheeler AP, Bernard GR, Thompson BT, Hayden D, de Boisblanc B, et al. Comparison of two fluid-management strategies in acute lung injury. N Engl J Med. 2006;354(24):2564–75. 16714767 10.1056/NEJMoa062200

[17] National Heart, Lung, and Blood Institute Acute Respiratory Distress Syndrome (ARDS) Clinical Trials Network, Matthay MA, Brower RG, Carson S, Douglas IS, Eisner M, Hite D, et al. Randomized, placebo-controlled clinical trial of an aerosolized β₂-agonist for treatment of acute lung injury. Am J Respir Crit Care Med. 2011;184(5):561–8. 21562125 10.1164/rccm.201012-2090OC PMC3175548

[18] National Heart, Lung, and Blood Institute Acute Respiratory Distress Syndrome (ARDS) Clinical Trials Network, Rice TW, Wheeler AP, Thompson BT, Steingrub J, Hite RD, Moss M, et al. Initial trophic vs full enteral feeding in patients with acute lung injury: the EDEN randomized trial. JAMA. 2012;307(8):795–803. 22307571 10.1001/jama.2012.137 PMC3743415

[19] National Heart, Lung, and Blood Institute ARDS Clinical Trials Network, Truwit JD, Bernard GR, Steingrub J, Matthay MA, Liu KD, Albertson TE, et al. Rosuvastatin for sepsis-associated acute respiratory distress syndrome. N Engl J Med. 2014;370(23):2191–200. 24835849 10.1056/NEJMoa1401520 PMC4241052

[20] Rizopoulos D. Joint models for longitudinal and time-to-event data: with applications in R. Boca Raton: CRC Press; 2012.

[21] Rizopoulos D, Ghosh P. A Bayesian semiparametric multivariate joint model for multiple longitudinal outcomes and a time-to-event. Stat Med. 2011;30:1366–80. 21337596 10.1002/sim.4205

[22] Rizopoulos D, Papageorgiou G, Afonso PM. JMbayes2: Extended joint models for longitudinal and time‐to‐event data. 2023. https://drizopoulos.github.io/JMbayes2/

[23] Rizopoulos D. The R package JMbayes for fitting joint models for longitudinal and time-to- event data using MCMC. J Stat Softw. 2016;72:46.

[24] Siddiki H, Kojicic M, Li G, Yilmaz M, Thompson TB, Hubmayr RD, et al. Bedside quantification of dead-space fraction using routine clinical data in patients with acute lung injury: secondary analysis of two prospective trials. Crit Care. 2010;14(4):R141. 20670411 10.1186/cc9206 PMC2945122

[25] Laffey JG, O’Croinin D, McLoughlin P, Kavanagh BP. Permissive hypercapnia–role in protective lung ventilatory strategies. Intensive Care Med. 2004;30(3):347–56. 14722644 10.1007/s00134-003-2051-1

[26] Laffey JG, Tanaka M, Engelberts D, Luo X, Yuan S, Tanswell AK, et al. Therapeutic hypercapnia reduces pulmonary and systemic injury following in vivo lung reperfusion. Am J Respir Crit Care Med. 2000;162(6):2287–94. 11112153 10.1164/ajrccm.162.6.2003066

[27] Ijland MM, Heunks LM, van der Hoeven JG. Bench-to-bedside review: hypercapnic acidosis in lung injury–from ‘permissive’ to ‘therapeutic.’ Crit Care. 2010;14(6):237. 21067531 10.1186/cc9238 PMC3220022

[28] Marhong J, Fan E. Carbon dioxide in the critically ill: too much or too little of a good thing? Respir Care. 2014;59(10):1597–605. 25261559 10.4187/respcare.03405

[29] Verscheure S, Massion PB, Verschuren F, Damas P, Magder S. Volumetric capnography: lessons from the past and current clinical applications. Crit Care. 2016;20(1):184. 27334879 10.1186/s13054-016-1377-3 PMC4918076

[30] Ferluga M, Lucangelo U, Blanch L. Dead space in acute respiratory distress syndrome. Ann Transl Med. 2018;6(19):388. 30460262 10.21037/atm.2018.09.46 PMC6212367

[31] Kaku N, Nakagama Y, Shirano M, Shinomiya S, Shimazu K, Yamazaki K, et al. Longitudinal ventilatory ratio monitoring for COVID-19: its potential in predicting severity and assessing treatment response. Crit Care. 2021;25(1):366. 34670589 10.1186/s13054-021-03768-2 PMC8527974

[32] Monteiro ACC, Vangala S, Wick KD, Delucchi KL, Siegel ER, Thompson BT, NHLBI PETAL Network, et al. The prognostic value of early measures of the ventilatory ratio in the ARDS ROSE trial. Crit Care. 2022;26(1):297. 36175982 10.1186/s13054-022-04179-7 PMC9521854

[33] Morales-Quinteros L, Schultz MJ, Bringué J, Calfee CS, Camprubí M, Cremer OL, MARS Consortium, et al. Estimated dead space fraction and the ventilatory ratio are associated with mortality in early ARDS. Ann Intensive Care. 2019;9(1):128. 31754866 10.1186/s13613-019-0601-0 PMC6872683

