# Supplementary material for: Time-varying intensity of ventilatory inefficiency and mortality in patients with acute respiratory distress syndrome
Source: Ann Intensive Care. 2025 Jan 13;15:6. doi: 10.1186/s13613-025-01427-1 (PMC11729588; doi:10.1186/s13613-025-01427-1)
Supplement: Supplementary file 1 — Additional file 1:A. Additional Methods: 1. Data collection. 2. Approach to missing data. 3. Bayesian joint model. B. Additional Results: Table S1 Characteristics of enrolled clinical trials. Table S2 Percentage of missing data in the baseline and dynamic cohort. Table S3 Adjusted hazard ratios of cause-specific Cox proportional model at baseline. Table S4 Baseline characteristics of patients stratified by PaCO2. Table S5 Baseline characteristics of patients stratified by ventilatory ratio. Table S6 The effect of cumulative exposure to hypercapnia or high VR on mortality. Table S7 The effect of time-varying exposure to hypercapnia or high VR after excluding missing data. Fig. S1 Directed acyclic graph illustrating the potential confounders on the association of ventilatory parameters and mortality. Fig. S2 Correlation matrix for baseline (A) and dynamic (B) covariates. Fig. S3 Study flow diagram in the present study. Fig. S4 Diagnostic plot of the joint model based on PaCO2. Fig. S5 Diagnostic plot of the joint model based on ventilatory ratio [file 13613_2025_1427_MOESM1_ESM.docx]

**Additional File 1**

This supplement provides additional information on methods and results, organized as follows:

1. **Additional methods:**
2. Data collection
3. Approach to missing data
4. Bayesian joint model
5. **Additional results:**

**Table S1** Characteristics of enrolled clinical trials

**Table S2** Percentage of missing data in the baseline and dynamic cohort

**Table S3** Adjusted hazard ratios of cause-specific Cox proportional model at baseline

**Table S4** Baseline characteristics of patients stratified by PaCO_2_

**Table S5** Baseline characteristics of patients stratified by ventilatory ratio

**Table S6** The effect of cumulative exposure to hypercapnia or high VR on mortality

**Table S7** The effect of time-varying exposure to hypercapnia or high VR after excluding missing data

**Fig. S1** Directed acyclic graph illustrating the potential confounders on the association of ventilatory parameters and mortality

**Fig. S2** Correlation matrix for baseline (A) and dynamic (B) covariates

**Fig. S3** Study flow diagram in the present study

**Fig. S4** Diagnostic plot of the joint model based on PaCO_2_

**Fig. S5** Diagnostic plot of the joint model based on ventilatory ratio

1. **Additional methods:**
2. **Data Collection**

Eligible patients in the ARDSNet trials were intubated and received positive-pressure ventilation, had a PaO_2_/FiO_2_ of less than 300 (adjusted if the altitude exceeded 1000 m), and had bilateral infiltrates on chest radiography consistent with the presence of pulmonary edema without evidence of left atrial hypertension. All patients must be randomized within 48 hours of meeting inclusion criterion. Following randomization, the low tidal volume protocol for mechanical ventilation must be initiated within one hour (if not already being utilized). Details of each trials were described in Additional file: Table S1. Longitudinal data of preselected variables including inspired fraction of oxygen (FiO_2_), arterial oxygen partial pressure (PaO_2_), positive end–expiratory pressure (PEEP), Plateau pressure (Pplat), peak pressure (Ppeak), tidal volume, minute ventilation and arterial carbon dioxide pressure (PaCO_2_) were obtained. After data collection, we made some calculations based on the following formulas:

- PaO_2_/FiO_2_= arterial oxygen partial pressure / inspired fraction of oxygen
- Ventilatory ratio= (minute ventilation* PaCO_2_) / ( PBW*100* 37.5)
- Driving pressure= Plateau Pressure – positive end expiratory pressure
- Compliance of the respiratory system= tidal volume / driving pressure

All the longitudinal variables were obtained from Day 0 until death, ICU discharge, liberation from mechanical ventilation or Day 28 in the ICU, whichever occurred first. We transformed all the longitudinal values <0 into missingness since these physiologically improbable values were assumed to be erroneous. The capping method was also applied to mitigate outlier influence.

1. **Approach to missing Data**

Missing data of the baseline and dynamic cohort was summarized in Tables S2. Prior to cox and joint model, we assumed that missing data was “missing at random”. Multiple imputation by chained equation (MICE) which generated values for all missing data using the observed data for all patients was applied through ‘mice’ package in R studio [1]. In total, we imputed 5 different datasets (n=5), the imputation method was weighted predictive mean matching.

1. **Bayesian joint model**

Bayesian joint models utilizes bayesian inference principles to effectively model the correlation between repeated measures and event times. By incorporating random effects, these models accommodate the inter-individual variability and inherent correlation within subject-specific trajectories [2]. The Bayesian framework facilitates the incorporation of prior information and uncertainty quantification, yielding posterior distributions for model parameters. This probabilistic approach is particularly advantageous in handling complex data structures and providing comprehensive insights into the dynamic relationship between longitudinal variables and associated event risks.

A joint longitudinal model consists of a mixed‐effects and a time‐to‐event submodel. Joint models add the subject-specific linear predictor of the mixed model as a time-varying covariate in the survival relative risk model. Because the model is complex, we kept increasing the number of Markov Chain Monte Carlo (MCMC) iterations until the MCMC chain demonstrates effective convergence towards the stationary distribution. Standard MCMC diagnostics of convergence include traceplot, density plot and potential scale reduction factor (Rhat) as close as to 1 [3].

Example R codes on how our joint model were implemented are listed below [4]:

**References:**

1. Zhang Z: Multiple imputation with multivariate imputation by chained equation (MICE) package. Ann Transl Med 2016, 4(2):30.
2. Rizopoulos D. Joint models for longitudinal and time‐to-event data: With Applications in R. CRC Press. 2012.
3. Rizopoulos D. The R package JMbayes for fitting joint models for longitudinal and time-to-event data using MCMC. J Stat Softw 2016; 72: 46.
4. Rizopoulos D, Papageorgiou G, Afonso PM. JMbayes2: Extended joint models forlongitudinal and time‐to‐event data. 2023. https://drizopoulos.github.io/JMbayes2/
5. **Additional results:**

**Table S1** Characteristics of enrolled clinical trials

|  | **FACTT** | **ALTA** | **EDEN** | **SAILS** |
| --- | --- | --- | --- | --- |
| **No. of patients** | 1000 | 282 | 1000 | 745 |
| **Enrollment period** | 2000-2005 | 2007-2008 | 2008-2011 | 2010-2013 |
| **Full title** | Fluids and Catheter Treatment Trial | Albuterol for the Treatment of ALI | Early versus Delayed Enteral Nutrition | Statins for Acutely Injured Lungs from Sepsis |
| **Inclusion criteria** | Patients with ARDS and received  invasive mechanical ventilation.  (AECC definition) | Patients with ARDS and received  invasive mechanical ventilation.  (AECC definition) | Patients with ARDS and received invasive mechanical ventilation.  (AECC definition) | Patients with ARDS and received invasive mechanical ventilation.  (AECC definition) |
| **Primary intervention** | Pulmonary Artery Catheters vs central venous catheter  Conservative  vs Liberal Fluid Management | Aerosolized Albuterol vs. Placebo | Initial lower-volume Trophic  vs Full Enteral Feeding | Rosuvastatin  vs Placebo |
| **Primary outcome** | 60-day mortality | Ventilator-free days to 28 days | Ventilator-free days to 28 days | 60-day mortality |

**Table S2** Percentage of missing data in the baseline and dynamic cohort

| **Variables** | **Baseline** | **Dynamic** |
| --- | --- | --- |
| Age | 0% | — |
| Gender | 0% | — |
| BMI | 2.95% | — |
| PBW | 0% | — |
| APACHE III score | 3.37% | — |
| ARDS primary risk factor | 0% | — |
| Solid tumor with metastasis | 1.16% | — |
| Immune suppression | 1.16% | — |
| Hepatic failure | 1.16% | — |
| Cirrhosis | 1.23% | — |
| Diabetes Mellitus | 1.19% | — |
| Hypertension | 6.07% | — |
| Congestive heart failure | 5.96% | — |
| Chronic pulmonary disease | 6% | — |
| pH | 2.88% | — |
| SPO_2_ | 2.35% | — |
| Set respiratory rate | 10.10% | — |
| Total respiratory rate | 0.84% | — |
| Tidal volume, mL/kg PBW | 16.31% | — |
| FiO_2_ | 0.49% | 8.96% |
| Minute ventilation | 3.61% | 10.96% |
| PEEP | 1.19% | 8.17% |
| Plateau Pressure | 32.59% | 37.01% |
| Peak inspiratory pressure | 8.21% | 19.70% |
| Driving pressure | 32.73% | 37.51% |
| Compliance | 35.64% | 41.61% |
| PaO_2_ | 2.88% | 22.21% |
| PaCO_2_ | 2.88% | 22.20% |
| PaO_2_/FiO_2_ ratio | 2.95% | 23.93% |
| Ventilatory ratio | 6.24% | 25.87% |
| Fluid balance | 1.37% | — |
| Vasopressors or inotropes use | 0% | — |
| Sedatives or NMBAs use | 0.11% | — |
| Ventilator-free days | 0.25% | — |

BMI body mass index; PBW predicted body weight; APACHE Acute Physiology and Chronic Health Evaluation; ARDS acute respiratory distress syndrome; pH potential of hydrogen; SpO_2_ peripheral capillary oxygen saturation; FiO_2_ inspired fraction of oxygen; PEEP positive end-expiratory pressure; PaO_2_ partial pressure of oxygen; PaCO_2_ arterial carbon dioxide pressure; PaO_2_/FiO_2_ ratio of arterial oxygen partial pressure to inspired fraction of oxygen；NMBAs neuromuscular blocking agents.

**Table S3** Adjusted hazard ratios of cause-specific Cox proportional model at baseline

| **Variables** | **Multivariate analysis** | | | |
| --- | --- | --- | --- | --- |
|  | **AHR (95%CI)** | **P Value** | **AHR (95%CI)** | **P Value** |
| Age, years | 1.024 (1.019-1.030) | <0.001 | 1.025 (1.019-1.030) | <0.001 |
| Gender | 1.105 (0.940-1.299) | 0.228 | 1.107 (0.942-1.301) | 0.217 |
| BMI | 1.000 (0.990-1.012) | 0.948 | 1.001 (0.990-1.011) | 0.900 |
| APACHE III | 1.019 (1.016-1.022) | <0.001 | 1.019 (1.016-1.022) | <0.001 |
| Comorbidities |  |  |  |  |
| Hypertension | 0.855 (0.712-1.027) | 0.094 | 0.856 (0.713-1.028) | 0.096 |
| Diabetes Mellitus | 0.852 (0.694-1.046) | 0.126 | 0.854 (0.696-1.049) | 0.133 |
| Chronic pulmonary disease | 1.154 (0.908-1.468) | 0.242 | 1.140 (0.899-1.445) | 0.280 |
| Tidal volume, mL/kg PBW | 1.004 (0.984-1.024) | 0.711 | 0.960 (0.918-1.003) | 0.071 |
| Use of sedatives or NMBAs | 0.595 (0.473-0.748) | <0.001 | 0.592 (0.471-0.745) | <0.001 |
| use of vasopressors or inotropes | 1.036 (0.870-1.233) | 0.692 | 1.037 (0.871-1.234) | 0.684 |
| PEEP | 1.019 (0.995-1.043) | 0.120 | 1.014 (0.990-1.039) | 0.247 |
| PaO_2_/FiO_2_ | 0.999 (0.998-1.000) | 0.043 | 0.999 (0.998-1.000) | 0.057 |
| PaCO_2_ | 1.003 (0.995-1.011) | 0.450 | — |  |
| Ventilatory ratio | — |  | 1.101 (1.009-1.201) | 0.031 |

AHR adjusted hazard ratio; CI confidence interval; BMI body mass index; APACHE Acute Physiology and Chronic Health Evaluation; PBW predicted body weight; NMBAs neuromuscular blocking agents; PEEP positive end-expiratory pressure; PaO_2_/FiO_2_ ratio of arterial oxygen partial pressure to inspired fraction of oxygen; PaCO_2_ arterial carbon dioxide pressure.

**Table S4** Baseline characteristics of patients stratified by PaCO_2_

| **Characteristics** | **Hypocapnia**  **(n=820)** | **Normocapnia**  **(n=1607)** | **Hypercapnia (n=342)** | **P value** |
| --- | --- | --- | --- | --- |
| Age, years | 53 [42,64] | 52 [40,63] | 49 [40,58] | 0.001 |
| Male , no (%) | 410 (48.9) | 855 (53.2) | 182 (53.2) | 0.117 |
| BMI | 27.1 [22.9,32.1] | 28.3 [23.9,34.0] | 30.3 [24.2,35.8] | <0.001 |
| APACHE III score | 96 [75,117] | 88 [70,108] | 93 [73,116] | <0.001 |
| Primary risk factor of ARDS—no (%) |  |  |  |  |
| Trauma | 27 (3.3) | 100 (6.2) | 7 (2.0) | <0.001 |
| Sepsis | 193 (23.5) | 311 (19.4) | 50 (14.6) | 0.001 |
| Multiple Transfusion | 9 (1.1) | 23 (1.4) | 1 (0.3) | 0.203 |
| Aspiration | 89 (10.9) | 187 (11.6) | 37 (10.8) | 0.809 |
| Pneumonia | 468 (57.1) | 907 (56.4) | 227 (66.4) | 0.003 |
| Others | 37 (4.5) | 83 (5.2) | 20 (5.8) | 0.609 |
| Comorbidities—no (%) |  |  |  |  |
| Solid tumor with metastasis | 13 (1.6) | 42 (2.7) | 6 (1.8) | 0.211 |
| Immune suppression | 90 (11.1) | 166 (10.5) | 42 (12.4) | 0.571 |
| Hepatic failure | 14 (1.7) | 11 (0.7) | 4 (1.2) | 0.066 |
| Cirrhosis | 47 (5.8) | 63 (4.0) | 16 (4.7) | 0.137 |
| Diabetes Mellitus | 211 (26.0) | 343 (21.7) | 71 (21.0) | 0.041 |
| Hypertension | 322 (41.9) | 623 (41.3) | 120 (37.6) | 0.400 |
| Congestive heart failure | 35 (4.6) | 75 (5.0) | 29 (9.1) | 0.006 |
| Chronic pulmonary disease | 55 (7.2) | 177 (11.7) | 67 (21.0) | <0.001 |
| Ventilator settings |  |  |  |  |
| FiO_2_, % | 0.5 [0.4,0.7] | 0.6 [0.5,0.7] | 0.6 [0.5,0.9] | <0.001 |
| Set respiratory rate, min^-1^ | 20 [16,26] | 20 [16,26] | 24 [18,30] | <0.001 |
| Total respiratory rate, min^-1^ | 26 [20,32] | 24 [20,30] | 25 [20,32] | <0.001 |
| Total minute ventilation, L/min | 11.9 [9.6,14.8] | 10.7 [8.9,13.1] | 10.1 [8.1,12.5] | <0.001 |
| Tidal volume, mL/kg PBW | 6.9 [6.0,8.0] | 6.6 [6.0,7.7] | 6.1 [5.9,7.0] | <0.001 |
| PEEP, cmH_2_O | 8 [5,10] | 10 [7,12] | 10 [8,14] | <0.001 |
| Plateau Pressure, cmH_2_O | 23 [19,28] | 24 [20,28] | 27 [22,31] | <0.001 |
| Peak Inspiratory Pressure, cmH_2_O | 27 [22,33] | 29 [24,35] | 32 [27,37] | <0.001 |
| Driving pressure, cmH_2_O | 14 [11,18] | 14 [11,19] | 16 [12,19] | 0.014 |
| Compliance | 30.0 [23.5,40.0] | 30.0 [22.5,39.2] | 25.2 [19.2,32.6] | <0.001 |
| Gas exchange |  |  |  |  |
| Arterial pH | 7.40 [7.34,7.45] | 7.37 [7.32,7.42] | 7.29 [7.22,7.35] | <0.001 |
| SpO_2_, % | 94 [70,98] | 95 [80,98] | 93 [82,98] | 0.029 |
| PaO_2_, mmHg | 82 [68,105] | 82 [69,103] | 77 [64,98] | 0.004 |
| PaCO_2_ , mmHg | 31 [28,33] | 41 [37,44] | 57 [53,64] | <0.001 |
| PaO_2_/FiO_2_, mmHg | 170 [120,227] | 150 [108,206] | 123 [86,185] | <0.001 |
| Ventilatory ratio | 1.6 [1.2,1.9] | 1.9 [1.5,2.3] | 2.6 [2.0,3.2] | <0.001 |
| Intervention |  |  |  |  |
| Fluid balance, mL | 2538 [810,4597] | 1792 [361,3619] | 1660 [304,3793] | <0.001 |
| Sedatives or NMBAs use, no (%) | 724 (88.4) | 1436 (89.4) | 316 (92.7) | 0.095 |
| Vasopressors or inotropes use, no (%) | 419 (51.1) | 685 (42.6) | 148 (43.3) | <0.001 |

Data are expressed as n (%) and median (interquartile range).

PaCO_2_ arterial carbon dioxide pressure; BMI body mass index; APACHE Acute Physiology and Chronic Health Evaluation; ARDS acute respiratory distress syndrome; FiO_2_ inspired fraction of oxygen; PBW predicted body weight; PEEP positive end-expiratory pressure; pH potential of hydrogen; SpO_2_ peripheral capillary oxygen saturation; PaO_2_ partial pressure of oxygen; PaO_2_/FiO_2_ ratio of arterial oxygen partial pressure to inspired fraction of oxygen；NMBAs neuromuscular blocking agents.

**Table S5** Baseline characteristics of patients stratified by ventilatory ratio

| **Characteristics** | **VR ≤ 2**  **(n=1646)** | **VR > 2**  **(n=1027)** | **P value** |
| --- | --- | --- | --- |
| Age, years | 50 [41,64] | 50 [40,61] | 0.001 |
| Male , no (%) | 948 (57.6) | 438 (42.6) | <0.001 |
| BMI | 27.8 [23.3,32.9] | 28.9 [24.0,35.0] | <0.001 |
| APACHE III score | 88 [70,108] | 95 [75,118] | <0.001 |
| Primary risk factor of ARDS—no (%) |  |  |  |
| Trauma | 104 (6.3) | 28 (2.7) | <0.001 |
| Sepsis | 349 (21.2) | 181 (17.6) | 0.027 |
| Multiple Transfusion | 25 (1.5) | 7 (0.7) | 0.080 |
| Aspiration | 200 (12.2) | 103 (10.0) | 0.105 |
| Pneumonia | 889 (54.0) | 659 (64.2) | <0.001 |
| Others | 83 (5.0) | 52 (5.1) | 1.000 |
| Comorbidities—no (%) |  |  |  |
| Solid tumor with metastasis | 37 (2.3) | 24 (2.4) | 1.000 |
| Immune suppression | 180 (11.1) | 108 (10.6) | 0.730 |
| Hepatic failure | 21 (1.3) | 7 (0.7) | 0.196 |
| Cirrhosis | 86 (5.3) | 37 (3.6) | 0.057 |
| Diabetes Mellitus | 404 (24.9) | 203 (19.9) | 0.003 |
| Hypertension | 658 (43.0) | 368 (37.7) | 0.010 |
| Congestive heart failure | 90 (5.9) | 45 (4.6) | 0.198 |
| Chronic pulmonary disease | 160 (10.4) | 125 (12.8) | 0.081 |
| Ventilator settings |  |  |  |
| FiO_2_, % | 0.5 [0.4,0.7] | 0.6 [0.5,0.8] | <0.001 |
| Set respiratory rate, min^-1^ | 20 [18,27] | 25 [20,30] | <0.001 |
| Total respiratory rate, min^-1^ | 22 [18,27] | 30 [25,35] | <0.001 |
| Total minute ventilation, L/min | 9.8 [8.1,11.9] | 13.1 [10.9,15.7] | <0.001 |
| Tidal volume, mL/kg PBW | 6.5 [6.0,7.6] | 6.7 [6.0,7.9] | 0.002 |
| PEEP, cmH_2_O | 8 [5,10] | 10 [8,12] | <0.001 |
| Plateau Pressure, cmH_2_O | 23 [19,27] | 27 [22,30] | <0.001 |
| Peak Inspiratory Pressure, cmH_2_O | 28 [22,33] | 31 [25,37] | <0.001 |
| Driving pressure, cmH_2_O | 14 [11,18] | 16 [12,20] | <0.001 |
| Compliance | 31.8 [24.7,41.1] | 25.7 [20.0,34.6] | <0.001 |
| Gas exchange |  |  |  |
| Arterial pH | 7.38 [7.33,7.43] | 7.34 [7.27,7.40] | <0.001 |
| SpO_2_, % | 95 [70,98] | 94 [88,98] | 0.850 |
| PaO_2_, mmHg | 84 [70,108] | 78 [66,97] | <0.001 |
| PaCO_2_ , mmHg | 36 [32,42] | 43 [37,50] | <0.001 |
| PaO_2_/FiO_2_, mmHg | 168 [123,225] | 127 [90,181] | <0.001 |
| Ventilatory ratio | 1.5 [1.3,1.8] | 2.5 [2.2,2.9] | <0.001 |
| Intervention |  |  |  |
| Fluid balance, mL | 2022 [516,4084] | 1895 [405,3930] | 0.233 |
| Sedatives or NMBAs use, no (%) | 1467 (89.3) | 929 (90.5) | 0.366 |
| Vasopressors or inotropes use, no (%) | 755(45.9) | 457 (44.5) | 0.514 |

Data are expressed as n (%) and median (interquartile range).

VR ventilatory ratio; BMI body mass index; APACHE Acute Physiology and Chronic Health Evaluation; ARDS acute respiratory distress syndrome; FiO_2_ inspired fraction of oxygen; PBW predicted body weight; PEEP positive end-expiratory pressure; pH potential of hydrogen; SpO_2_ peripheral capillary oxygen saturation; PaO_2_ partial pressure of oxygen; PaCO_2_ arterial carbon dioxide pressure; PaO_2_/FiO_2_ ratio of arterial oxygen partial pressure to inspired fraction of oxygen; NMBAs neuromuscular blocking agents.

**Table S6** The effect of cumulative exposure to hypercapnia or high VR on mortality

|  | **Model with PaCO_2_** | |  | **Model with VR** | |
| --- | --- | --- | --- | --- | --- |
|  | HR (95% CI) | P value |  | HR (95% CI) | P value |
| **Baseline characteristics** |  | |  | | |
| Age, years | 1.023(1.016-1.031) | <0.001 |  | 1.023(1.016-1.031) | <0.001 |
| Gender | 1.098(0.926-1.293) | 0.272 |  | 1.118(0.943-1.320) | 0.188 |
| BMI | 1.000(0.989-1.010) | 0.907 |  | 1.000(0.989-1.010) | 0.999 |
| APACHE III | 1.020(1.017-1.023) | <0.001 |  | 1.019(1.017-1.023) | <0.001 |
| Hypertension | 0.863(0.711-1.036) | 0.117 |  | 0.863(0.721-1.045) | 0.129 |
| Diabetes Mellitus | 0.834(0.688-1.007) | 0.065 |  | 0.837(0.690-1.018) | 0.077 |
| Chronic pulmonary disease | 1.208(0.973-1.500) | 0.082 |  | 1.212(0.973-1.507) | 0.092 |
| Tidal volume, mL/kg PBW | 0.997 (0.965-1.015) | 0.970 |  | 0.995(0.961-1.015) | 0.856 |
| Positive end-expiratory pressure | 1.021(0.998-1.044) | 0.077 |  | 1.018(0.995-1.041) | 0.118 |
| Use of sedatives or NMBAs | 0.595(0.479-0.754) | <0.001 |  | 0.590 (0.475-0.744) | <0.001 |
| Use of vasopressors or inotropes | 1.027(0.862-1.219) | 0.757 |  | 1.042(0.876-1.244) | 0.666 |
| **Time-varying variables** |  |  |  |  |  |
| PaO_2_/FiO_2_ | 0.998(0.996-1.000) | 0.017 |  | 0.999(0.997-1.001) | 0.195 |
| Area with PaCO_2_>50,  (mmHg*day) | 1.002(0.999-1.004) | 0.199 |  |  |  |
| Area with VR>2,  (1*day) |  |  |  | 1.085(1.050-1.122) | <0.001 |

Number of subjects: 2851; Number of events: 608 (21.3%); Number of observations: 15989. The estimate represents the increase in the hazard of death for every 1-unit increase in the area below the longitudinal profile and above the indicated threshold for PaCO_2_ and ventilatory ratio. The area term therefore reflects the cumulative exposure to different doses of high ventilatory inefficiency. VR ventilatory ratio; HR hazard ratio; CI confidence interval; BMI body mass index; APACHE Acute Physiology and Chronic Health Evaluation; PBW predicted body weight; NMBAs neuromuscular blocking agents; PaO_2_/FiO_2_ ratio of arterial oxygen partial pressure to inspired fraction of oxygen; PaCO_2_ arterial carbon dioxide pressure.

**Table S7** The effect of time-varying exposure to hypercapnia or high VR after excluding missing data

|  | **Model with PaCO_2_** | |  | **Model with VR** | | |
| --- | --- | --- | --- | --- | --- | --- |
|  | HR (95% CI) | P value |  | HR (95% CI) | P value | |
| **Baseline characteristics** |  | |  | | |  |
| Age, years | 1.024(1.013-1.035) | <0.001 |  | 1.024(1.012-1.035) | <0.001 | |
| Gender | 0.997(0.780-1.264) | 0.987 |  | 1.018(0.787-1.320) | 0.892 | |
| BMI | 0.993(0.979-1.008) | 0.369 |  | 0.994(0.979-1.008) | 0.438 | |
| APACHE III | 1.019(1.015-1.023) | <0.001 |  | 1.018(1.014-1.022) | <0.001 | |
| Hypertension | 0.910(0.692-1.203) | 0.519 |  | 0.881(0.669-1.174) | 0.360 | |
| Diabetes Mellitus | 0.966(0.720-1.308) | 0.830 |  | 1.012(0.756-1.361) | 0.943 | |
| Chronic pulmonary disease | 1.573(1.106-2.183) | 0.015 |  | 1.622(1.168-2.271) | 0.004 | |
| Tidal volume, mL/kg PBW | 1.080(1.001-1.164) | 0.048 |  | 1.057(0.981-1.138) | 0.154 | |
| Positive end-expiratory pressure | 1.021(0.989-1.054) | 0.217 |  | 1.018 (0.985-1.049) | 0.272 | |
| Use of sedatives or NMBAs | 0.616(0.437-0.886) | 0.008 |  | 0.604(0.427-0.884) | 0.009 | |
| Use of vasopressors or inotropes | 1.220 (0.950-1.562) | 0.111 |  | 1.276 (0.992-1.640) | 0.057 | |
| **Time-varying variables** |  |  |  |  |  | |
| PaO_2_/FiO_2_ | 0.996(0.993–0.999) | 0.007 |  | 0.997 (0.993-1.000) | 0.028 | |
| Any exposure to hypercapnia  (PaCO_2_ >50mmHg) | 1.073(0.998-1.158) | 0.057 |  |  |  | |
| Any exposure to high VR  (Ventilatory ratio >2) |  |  |  | 1.088(1.021–1.164) | 0.009 | |

Number of subjects:1205; Number of events:309 (25.6%); Number of observations:6158. The hazard ratios were the adjusted hazard ratios associated with 1 increment in the given variable. VR ventilatory ratio; HR hazard ratio; CI confidence interval; BMI body mass index; APACHE Acute Physiology and Chronic Health Evaluation; PBW predicted body weight; NMBAs neuromuscular blocking agents; PaO_2_/FiO_2_ ratio of arterial oxygen partial pressure to inspired fraction of oxygen; PaCO_2_ arterial carbon dioxide pressure.

**Fig. S1** Directed acyclic graph illustrating the potential confounders on the association of ventilatory parameters and mortality****

ARDS acute respiratory distress syndrome; VR ventilatory ratio; BMI body mass index; APACHE Acute Physiology and Chronic Health Evaluation; FiO_2_ inspired fraction of oxygen; pbw predicted body weight; PEEP positive end-expiratory pressure; NMBAs neuromuscular blocking agents; pH potential of hydrogen; SpO_2_ peripheral capillary oxygen saturation; PaCO_2_ arterial carbon dioxide pressure; PaO_2_/FiO_2_ ratio of arterial oxygen partial pressure to inspired fraction of oxygen; Pplat plateau Pressure; △P driving pressure.

The minimal sufficient adjustment sets for estimating the total effect of Ventilatory parameters [PaCO2&VR] on 28-day mortality: age, Gender, BMI, APACHE, Combidities, PEEP, Tidal volume per pbw, Use of sedatives or NMBAs, Use of vasopressors or inotropes and PaO_2_/FiO_2_.

**Fig. S2** Correlation matrix for baseline(A) and dynamic(B) covariates

bmi body mass index; apache Acute Physiology and Chronic Health Evaluation; fio2 inspired fraction of oxygen; srate set respiratory rate; resp respiratory rate; tmnvnt minute ventilation; tidalpbw tidal volume per predicted body weight; peep positive end-expiratory pressure; pplat plateau pressure; pip peak inspiratory pressure; dp driving pressure; crs compliance of the respiratory system; ph potential of hydrogen; spo2 peripheral capillary oxygen saturation; pao2 partial pressure of oxygen; paco2 arterial carbon dioxide pressure; pf ratio of arterial oxygen partial pressure to inspired fraction of oxygen; vr ventilatory ratio; fb fluid balance.

**Fig. S3** Study flow diagram in the present study

ARDS: acute respiratory distress syndrome; PBW: predicted body weight.

**Fig. S4** Diagnostic plot of the joint model based on PaCO_2_. A. Traceplot. B. Density plot. PaCO_2_ arterial carbon dioxide pressure.

**Fig. S5** Diagnostic plot of the joint model based on ventilatory ratio. A.Traceplot. B. Density plot. vr ventilatory ratio.
